# Dynamic Nonlinear Spatial Integrations on Encoding Contrasting Stimuli of Tectal Neurons

**DOI:** 10.3390/ani14111577

**Published:** 2024-05-26

**Authors:** Shuman Huang, Pingge Hu, Zhenmeng Zhao, Li Shi

**Affiliations:** 1 Key Laboratory of Artificial Intelligence and Personalized Learning in Education of Henan Province, College of Computer and Information Engineering, Henan Normal University, Xinxiang 453007, China; 2 Department of Automation, Tsinghua University, Beijing 100084, China; hpg18@mails.tsinghua.edu.cn; 3 School of Software, Henan Normal University, Xinxiang 453007, China; zhaozhenmeng@htu.edu.cn

**Keywords:** dynamic nonlinear spatial integrations, surround modulation, optic tectum, contrasting stimuli

## Abstract

**Simple Summary:**

Animals need to identify important visual targets, like food or predators, from a busy background. This ability relies on how their visual system processes and highlights different visual cues. When it comes to birds, the neurons in the optic tectum play a crucial role in this process, particularly through mechanisms known as surround modulation. Our study used a computer computational model to explore how these neurons respond to different types of visual cues like changes in brightness and motion. We discovered that such a process involves complex, nonlinear interactions and inhibitory signals. These findings may help us better understand how birds and other animals detect and focus on important objects from the environment.

**Abstract:**

Animals detect targets using a variety of visual cues, with the visual salience of these cues determining which environmental features receive priority attention and further processing. Surround modulation plays a crucial role in generating visual saliency, which has been extensively studied in avian tectal neurons. Recent work has reported that the suppression of tectal neurons induced by motion contrasting stimulus is stronger than that by luminance contrasting stimulus. However, the underlying mechanism remains poorly understood. In this study, we built a computational model (called Generalized Linear-Dynamic Modulation) which incorporates independent nonlinear tuning mechanisms for excitatory and inhibitory inputs. This model aims to describe how tectal neurons encode contrasting stimuli. The results showed that: (1) The dynamic nonlinear integration structure substantially improved the accuracy (significant difference (*p* < 0.001, paired *t*-test) in the goodness of fit between the two models) of the predicted responses to contrasting stimuli, verifying the nonlinear processing performed by tectal neurons. (2) The modulation difference between luminance and motion contrasting stimuli emerged from the predicted response by the full model but not by that with only excitatory synaptic input (spatial luminance: 89 ± 2.8% (GL_DM) vs. 87 ± 2.1% (GL_DMexc); motion contrasting stimuli: 87 ± 1.7% (GL_DM) vs. 83 ± 2.2% (GL_DMexc)). These results validate the proposed model and further suggest the role of dynamic nonlinear spatial integrations in contextual visual information processing, especially in spatial integration, which is important for object detection performed by birds.

## 1. Introduction

Animals whose survival depends on early target detection are often equipped with a sharply tuned visual system, yielding robust performance in challenging conditions. Complex scene perception depends upon the interaction between signals from the classical receptive field (CRF) and the extra-classical receptive field (eCRF) in visual neurons [1]. Among them, there is a fundamental neural phenomenon known as “surround suppression”. This phenomenon has been widely reported to exist in many levels of the visual system, including the retina [2,3], superior colliculus (SC) [4], lateral geniculate nucleus (LGN) [5], and primary visual cortex (V1) [6,7], in mammals and the optic tectum (OT) [8,9,10] in non-mammals. This fundamental neuronal property is associated with visual saliency representation, allowing efficient information encoding. The electrophysiological experiments further show that the suppression degree could be modulated by stimuli contrast between the center and surroundings [11,12,13]. The suppression is strongest in homogeneous visual contexts, where the surrounding stimuli are similar to those in the center [14]. Recent work has reported that the suppression engendered by motion direction contrasting stimuli was stronger than that by static luminance contrasting stimuli [12], suggesting that encoding strategies of luminance and motion contrasting stimuli may be different. Furthermore, under the motion mode, the surround modulation index induced by luminance contrast is lower than that under the static mode (authors, unpublished data), suggesting that the surround modulation pattern of OT neurons is dynamic, not only related to the degree of contrast between the center and surroundings but also associated with the state of the target (stimulus mode). However, it is still unclear how they differ and where the difference was derived from. The answer would help with further understanding the underlying encoding mechanisms of surround suppression, which plays an important role in enhancing contrast sensitivity and effectively reducing redundant information in the visual environment.

Abundant existing studies imply that the neuronal processing of external visual information is always nonlinear and the tuning of excitatory and inhibitory inputs might also differ substantially [15,16]. However, nonlinear processing was always approximated with a linear module in encoding models [17,18] to reduce the modeling complexity and facilitate computational efficiency. Typically, the center–surround effect has been widely explained using a difference of Gaussian (DoG) model [2,19], which was defined by the difference of two Gaussian distributions with a narrower positive Gaussian and a broader negative Gaussian. This model assumes that the surround interaction is linear with the fully formed center signal. Furthermore, the generalized linear model (GLM) was always used to link the functional descriptions of visual processing with neural activity recorded across the visual system [20,21] and described the encoding process in terms of a series of stages: linear filtering, nonlinear transformation, and Poisson spiking process. Except, GLM was used to characterize neural encoding in a variety of other sensory, cognitive, and motor brain areas [17,21]. These existing models also hypothesized that the interaction between excitatory and inhibitory inputs was linear, whereas nonlinearity serves as a typical characteristic that drives the underlying circuitry and promotes sensory integration. It is unclear whether the linear modules instead of nonlinear modules can realistically simulate the encoding process of optic tectal neurons for moving objects.

Meanwhile, it could be implied that center–surround suppression is closely related to the suppression inputs that neurons receive, including those from feedforward, horizontal, and feedback connections [22]. Recent work also suggested that inhibitions performed on tectal neurons appeared to be fully surrounding rather than locally lateral [10]. Additionally, a saliency map in the avian optic tectum (OT) is created by the interaction between the OT and the nucleus isthmi. The latter is divided into two streams: the nucleus isthmi pars magnocellularis (Imc) and the nucleus isthmi pars parvocellularis (Ipc) [23], transmitting inhibitory and excitatory inputs into OT, respectively. Both reductions in excitatory synaptic input and an increase in inhibitory synaptic input lead to a decrease in total synaptic input, which could generate diverse modulation for different contrasting stimuli. However, there is a lack of evidence proving which of these inputs contributed to the diverse contextual modulated responses. Furthermore, it is pretty difficult to accurately describe the encoding process of a complex neuronal network, especially containing such nonlinearity and complex interactions. Fortunately, the conductance-based encoding model (CBEM), as an extension of GLM, could simulate synaptic conductance changes that drive them by not only allowing for differential tunings of excitation and inhibition but also adding rectifying nonlinearities governing the relationship between the stimulus and synaptic input [24]. The inhibitory and excitatory components could be flexibly adjusted in CBEM. Thus, it is well suited to study the encoding strategies on spatial contrasting stimuli.

Taken together, we hypothesized that diverse surround modulation properties could be regulated by dynamical nonlinear spatial integration between excitation and inhibitory synaptic inputs. Then, the CBEM framework was adopted to establish a contrasting encoding model (Generalized Linear-Dynamic Modulation, GL_DM) to describe the process of encoding contrasting stimuli in tectal neurons, and the parameters were fitted by neuronal data recorded from pigeon OT in the same stimulus paradigms with our previous work [12], including motion direction contrasting stimuli and luminance contrasting stimuli. The results show that the encoding accuracy was improved by taking the nonlinear functions into account, suggesting that the dynamic nonlinear spatial integrations may play an important role in encoding contrasting visual stimuli. Finally, we compared the predicted response by the full-GL_DM and GL_DM with only excitatory synaptic input to verify the factor that affects the modulation difference between luminance and motion contrasting stimuli.

## 2. Materials and Methods

### 2.1. The Control Model: The Generalized Linear Model Combined with the Difference of Gaussian Model

**Figure 1:**
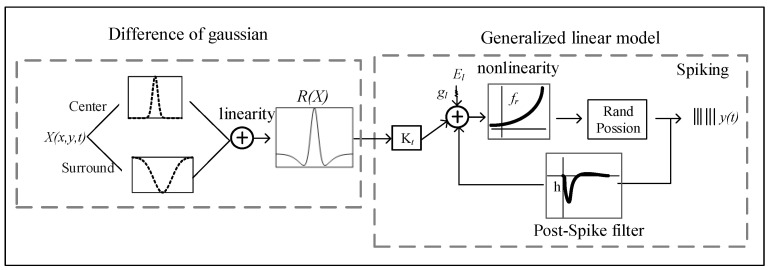
Schematic of the generalized linear model combined with the difference of Gaussian model (DoG), hereinafter referred to as GLM.

The generalized linear model combined with the difference of Gaussian model serves as a control linear model. The model schematic is shown in Figure 1. It mainly consists of two parts: the difference of Gaussian model and the generalized linear model. The former provides a mapping from stimuli to spike trains, and the latter describes the realistic center–surround receptive field (RF) structure.

The difference of Gaussian model represents the RF center–surround integration and consists of a positive narrow Gaussian function, used to describe the excitability of the receptive field center, and a negative broad Gaussian function to describe the inhibitory ingredients of antagonistic receptive field surroundings. The summation of them represents their mutual antagonism in space. The output is represented by the two Gaussian functions on the spatial domain after linear weighted integral. The function of the DoG filter is expressed as follows:

(1) $$\begin{array}{c} R\left(X\right)=K_{e}\cdot N(X,\mu ,r_{e})-K_{i}\cdot N(X,\mu ,r_{i})\\ N(X,\mu ,r)=e^{-{\left(\frac{X-\mu }{2*r}\right)}^{2}} \end{array}$$

where N(X,μ,r) is a Gaussian function with the mean value μ, and $r_{e}$ and $r_{i}$ indicate the size of excitatory RF and inhibitory surrounding RF, respectively. $k_{e}$ and $k_{i}$ denote the relative strength of excitability and inhibition, respectively. $k_{e}\geq k_{i}$ represents the weaker RF surround strength relative to the RF center. $R\left(X\right)$ represents the spatial integrated output between the center and the surroundings.

The generalized linear model is a cascade model, containing linear integration, the nonlinear transition of spike rate, and probabilistic spiking stages of the Poisson process. Spatiotemporal integration of visual inputs from cones is modeled as linear, in which spatial integration is a two-dimensional Gaussian function and temporal integration is a biphasic impulse-response function. The linear integration stage consists of spatiotemporal integration of visual inputs and post-spike filter, in which the former spatial integration of stimulus is described with the DoG model, and temporal integration of stimulus is characterized for a filter $K_{t}$ in GLM. The neuron’s membrane potential is defined as follows:

(2) $$V_{t}=K_{t}\cdot R(X)-h\cdot y_{t}^{hist}+b$$

where $R\left(X\right)$ is the spatially integrated stimulus (vectorized). $K_{t}$ is the stimulus temporal filter. h is the post-spike filter to capture dependencies on spike history. $y_{t}^{hist}$ is a vector representing spike history at time *t*. b is a baseline to determine the baseline firing rate in the absence of input.

Then, membrane potential passes through a nonlinear function $f_{r}$ to produce the spike rate $\lambda_{t}$ as the conditional intensity of a Poisson process.

(3) $$\lambda_{t}=f_{r}\left(V_{t}\right)$$

Finally, the spike trains are obtained by an inhomogeneous Poisson spiking process.

(4) $$y_{t}\left|\lambda_{t}\right.∼Poiss\left(△\lambda_{t}\right)$$

### 2.2. Contrasting Encoding Model: Generalized Linear-Dynamic Modulation

**Figure 2:**
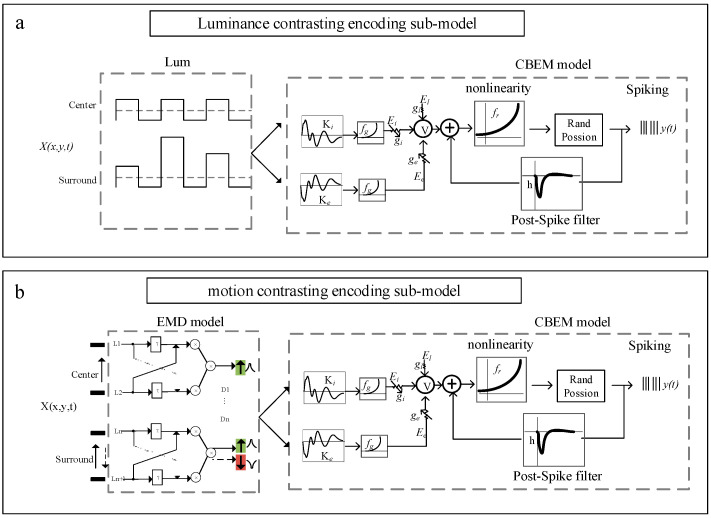
(**a**) Schematic of the luminance contrasting encoding sub-model. The contrasting encoding model (referred to as GL_DM) mainly consists of two parts: the photoelectric transduction model and the conductance-based encoding model (CBEM). (**b**) Schematic of the motion direction contrasting encoding sub-model. The arrow direction indicates direction of movement. The green and red arrows indicate that the direction of movement is opposite.

A contrasting encoding model (referred to as GL_DM) is established based on the CBEM framework cascading with a photoelectric transduction model to describe the primary process of encoding contrasting stimuli. In contrast to the GLM, CBEM adds a nonlinear governing relationship between the stimulus and synaptic conductance with independent tunings of excitatory and inhibitory synaptic inputs. GL_DM mainly consists of two parts: the photoelectric transduction model and the CBEM. The former is used to simulate the preprocessing of temporal and spatial stimuli by the retina and tectum, and the latter is used to generate spike trains of OT neurons to specific stimuli. The GL_DM schematic for predicting spike trains of tectal neurons for contrasting stimuli is shown in Figure 2.

The GL_DM was established to validate encoding strategies by exploring key factors that contribute to the visual response differences between two different types of stimuli (spatial luminance contrasting versus motion direction contrasting). Thus, the inputs of the model contain two different types of visual stimuli (static and moving stimuli), which are processed by two types of photoelectric transduction modules, respectively. As is shown in the green region in Figure 2a, the static visual stimulus is transduced to electrical activity by normalization of luminance values. At time t, the electrical activity with a range of [−1 1] at a single point in space is defined as follows:

(5) $$X\left(t\right)=\frac{x-\bar{X}}{X_{\max}-X_{\min}}$$

where x is the luminance value at a single point in space. $\bar{X}$ is the mean luminance value. $X_{\max}$ and $X_{\min}$ indicate the maximum and minimum luminance values, respectively.

In contrast to static stimulus, the electrical activity in each spatial pixel is time-varying for moving stimulus (Figure 2b). The direction of motion stimuli is detected by the full Hassenstein–Reichardt detector (full-HR detector), which consists of two subunits, arranged in a mirror-symmetric fashion and subtracted from each other to yield a fully opponent detector. The output $X\left(t\right)$ is defined as follows:

(6) $$X\left(t\right)=\left[L_{1}\left(t\right)*L_{2}\left(t-\tau \right)\right]-\left[L_{1}\left(t-\tau \right)*L_{2}\left(t\right)\right]$$

where $L_{1}\left(t\right)$ and $L_{2}\left(t\right)$ are two adjacent dendrites to detect the contrast signals from two spatial locations. τ denotes the temporal delay.

As a result, the sign of the output is positive for the preferred direction, and negative when motion is along the opposite direction. The preferred direction was defined as the motion direction of the stimulus inside RF since the neurons in this study had no motion direction selectivity [12].

By using the photoelectric transduction model, the stimulus is transduced as a matrix, serving as the input of CBEM, which is an extension of GLM by adding two linear–nonlinear functions of input to obtain excitatory and inhibitory synaptic conductance, respectively.

(7) $$\begin{array}{l} g_{e}\left(t\right)=f_{g}\left(k_{e}\cdot X\left(t\right)+b_{e}\right)\\ g_{i}\left(t\right)=f_{g}\left(k_{i}\cdot X\left(t\right)+b_{i}\right) \end{array}$$

Here, $X\left(t\right)$ is the output of the photoelectric transduction model. $k_{e}$ and $k_{i}$ denote the linear excitatory and inhibitory “conductance” filter. The nonlinear function $f_{g}$ is assumed a “soft-rectification” to ensure that conductance is non-negative. $b_{e}$ and $b_{i}$ determine the baseline excitatory and inhibitory conductance in the resting state.

After giving excitatory and inhibitory synaptic conductance, the membrane potential $V_{t}$ is defined by an ordinary differential equation:

(8) $$\frac{dV_{t}}{dt}=-g_{l}\left(V_{t}-E_{l}\right)-g_{e}\left(t\right)\left(V_{t}-E_{e}\right)-g_{i}\left(t\right)\left(V_{t}-E_{i}\right)$$

where $g_{l}$ is leak conductance. $E_{l}$, $E_{e}$, and $E_{i}$ are the leak, excitatory, and inhibitory reversal potentials.

Then, the spike-history effect is incorporated by adding a linear autoregressive term:

(9) $${\tilde{V}}_{t}=V_{t}+h\cdot y_{t}^{hist}$$

where $y_{t}^{hist}$ is a vector of binned spike history at time *t*. *h* is the spike-history filter.

Finally, the outputs ${\tilde{V}}_{t}$ pass through a nonlinear function $f_{r}$ to obtain the spike rate for an inhomogeneous Poisson spiking process.

(10) $$\lambda \left(t\right)=f_{r}\left({\tilde{V}}_{t}\right)$$

(11) $$y_{t}/\lambda_{t}\sim Poiss\left(\Delta \lambda_{t}\right)$$

The model parameters needing to fit include $k_{e}$, $k_{i}$, $b_{e}$, $b_{i}$ and h, all of which are selected using conjugate-gradient methods to maximize the log likelihood. The reversal potential and leak conductance parameters [25,26] are kept fixed at $E_{e}=0\mathrm{mV}$, $E_{l}=-60\mathrm{mV}$, $E_{i}=-80\mathrm{mV}$, and $g_{l}=200$.

### 2.3. Electrophysiological Data

The electrophysiological data were selected from the previous work [12] and recorded from 10 pigeons (Columba livia, homing pigeon, either sex, weighing 300–400 g). The neuronal data were obtained with a polyimide-insulated platinum/iridium micro-electrode array that was arranged in four rows with four wires in each row. The array was lowered about 800 μm–1200 μm below the tectum surface using a micromanipulator.

Two types of center–surround stimulus paradigms were used in this study: one involving luminance stimuli and the other involving motion stimuli. In the luminance contrasting paradigm, a vertical or horizontal bar is placed at the center of the receptive field (RF). Surrounding bars of equal size are positioned parallel to the orientation of the central bar. The luminance of the center bar was set at 0.1 cd/m^2^. The luminance of the surrounding bars was uniformly set at 0.1 cd/m², 35.1 cd/m², and 70.1 cd/m². In the motion contrasting paradigm, the motion direction and luminance of the center bar were kept the same too. The surrounding bars moved in the same or opposite direction. The luminance of the surrounding bars was uniformly set at 0.1 cd/m² or 70.1 cd/m².

A total of 30 neurons were recorded, with 20 trials collected for each neuron. Fifteen neurons were used for the luminance stimulus paradigm, and nineteen neurons were used for the motion stimulus paradigm. Four neurons were used for both paradigms, resulting in a total of 680 data points.

Each set of parameters was fitted with neuronal responses to two types of center–surround stimuli, including flashed spatial luminance contrasting mode and motion direction contrasting mode with uniform luminance, in which the center bar was at RF center while the surrounding ones varied in luminance and motion direction were distributed outside the RF.

### 2.4. Evaluation of Model Performance

Firstly, we fit the model parameters to a dataset consisting of two types of center–surround stimuli and their corresponding firing spike trains, and then we used the trained model to predict the spike responses elicited in response to novel stimuli recorded in the same unit. The training set is divided using the 80:20 rule, where 80% of the recordings of each class separately are used for training, and the remaining are used for validation. The model performance was evaluated with the residual error (e(t)) and goodness of fit ($R^{2}$) between the predicted peri-stimulus time histogram (PSTH) and the recorded neuronal response. The PSTH was calculated by averaging spike numbers in 20 ms time bins with smoothing with a Gaussian filter. The residual error is defined as follows:

(12) $$e\left(t\right)=\left(\sum_{i=m-n}^{m}N_{i}-\int_{t_{m-n}}^{t_{m}}\lambda \left(t\right)\right)÷I$$

According to the nature of the residual, ideally the residual points of the model should be evenly distributed on the upper and lower sides of the y=0 straight line. The smaller the residual value, the higher the prediction accuracy of the model. The good-ness fit is defined as follows:

(13) $$R^{2}=\frac{\sum_{t=1}^{T}\left(PSTH_{\mathrm{Data}}\left(t\right)-\bar{PSTH_{\mathrm{Data}}}\right)\left(PSTH_{\mathrm{Model}}\left(t\right)-\bar{PSTH_{\mathrm{Model}}}\right)}{{\left\{\sum_{t=1}^{T}{\left(PSTH_{\mathrm{Data}}\left(t\right)-\bar{PSTH_{\mathrm{Data}}}\right)}^{2}\sum_{t=1}^{T}{\left(PSTH_{\mathrm{Model}}\left(t\right)-\bar{PSTH_{\mathrm{Model}}}\right)}^{2}\right\}}^{\frac{1}{2}}}$$

where $\bar{PSTH_{\mathrm{Model}}}$ and $\bar{PSTH_{\mathrm{Data}}}$ denote the average value of the PSTH. The value of the rho ranges from −1 to +1, where ±1 indicates a total positive or negative correlation and 0 indicates no correlation.

We also calculated a modulation index to verify whether the proposed model captured their observed surrounding modulation in experimental data. The modulation index is defined as follows: modulation index = (*R*_oddball_ − *R*_uniform_)/(*R*_oddball_ + *R*_uniform_), where *R*_oddball_ is the response to the center stimulus when it is different from the background elements, and *R*_uniform_ is the response to the same center stimulus when it is identical to the background elements.

All statistical tests performed in this manuscript were non-parametric Wilcoxon signed-rank tests, unless otherwise stated. Data analysis was performed using MATLAB R2019b (The Mathworks). Graphs and figures were performed using Origin 2019b (Origin Lab, Northampton, CA, USA).

## 3. Results

The model built in this paper was fitted and tested with the experimental data [12] consisting of spike trains in 15 recording sites for luminance contrasting stimuli and 19 recording sites for motion contrasting stimuli. This study employs a dynamic modulation encoding model based on dynamic nonlinear integration to predict the responses of tectal neurons under various contrasting stimuli. A comparison is made with the conventional generalized linear model based on Gaussian difference to demonstrate the superiority of dynamic nonlinear stimulus integration in characterizing the contrasting modulation patterns of tectal neurons. The detailed results of the one recoding site are presented together with the key results of other sites.

### 3.1. Nonlinear Integration on Encoding Contrasting Stimuli

**Figure 3:**
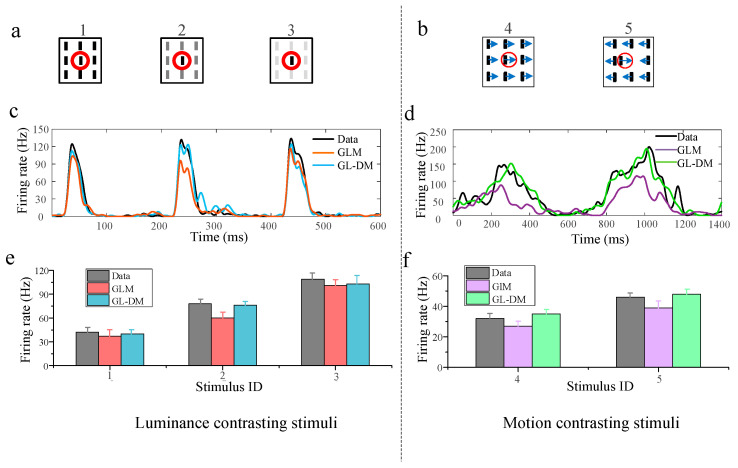
The predicted neuronal response by the computational model. (**a**) Luminance contrasting stimulus at three contrast levels. The red circle in each subfigure indicates the receptive field area for the example recording site. (**b**) Motion contrasting stimuli at two contrast levels. The red circle in each subfigure indicates the receptive field area for the example recording site. The Arrow direction indicates direction of movement. (**c**) The peri-stimulus time histogram (PSTH) for luminance contrasting stimulus from the raw data, GL_DM, and the GLM. (**d**) The peri-stimulus time histogram (PSTH) for motion contrasting stimuli from the raw data, GL_DM, and the GLM. (**e**) The statistical results of the mean firing rate to each stimulus are shown in (**a**). (**f**) The statistical results of the mean firing rate to each stimulus are shown in (**b**).

To examine the role of nonlinear integration in encoding contrasting stimuli, we compared the recorded data and predicted response (Figure 3). For the predicted response, one is derived from GLM using linear filtering the other is derived from GL_DM using nonlinear functions. The neuronal data were obtained from a total of five contrast levels in the paradigms, including three for luminance stimuli (Figure 3a) and two for motion stimuli (Figure 3b). Figure 3a,b presents different modes of visual stimuli, and the red solid line circle indicates the border of the RF. The original PSTHs of the different stimuli are presented in Figure 3c,d. The corresponding mean firing rate under each stimulus mode was calculated, and the statistical results of 20 repeats are shown in Figure 3e,f. The results between the recorded and predicted neuronal responses illustrated that, to a certain degree, both models could capture diverse contextual modulated response properties and that neuronal response increases as contrast increases.

**Figure 4:**
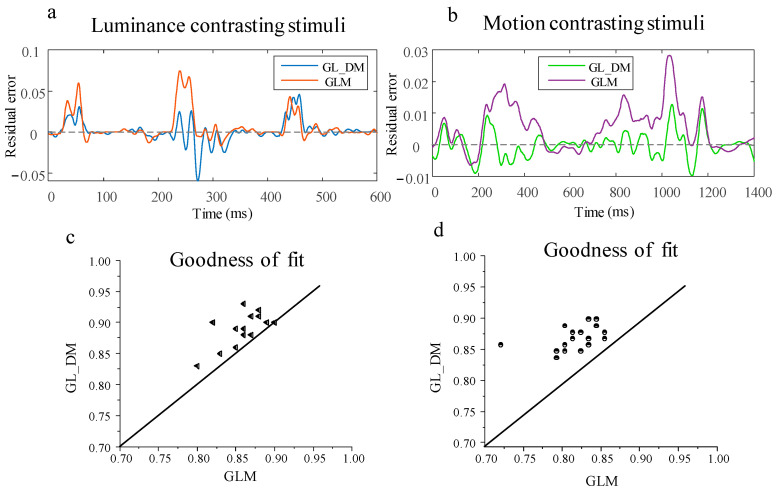
The residuals and goodness of fit for the computational model. (**a**) The residual plots for the GL_DM model and GLM model under luminance contrasting stimulus; (**b**) the residual plots for the GL_DM model and GLM model under motion contrasting stimuli; (**c**) goodness of fit plots for the GL_DM model and GLM model under luminance contrasting stimulus; (**d**) goodness of fit for the GL_DM model and GLM model under motion contrasting stimuli.

Furthermore, the statistical results of prediction residuals and goodness of fit for both models are depicted in Figure 4. The residuals of the two models are illustrated in Figure 4a,b. It is evident from the plots that the prediction residuals of both models are distributed around the line of zero residuals, indicating a good fit to the original observed values. However, the results obtained from the GL_DM model were slightly higher values than the recorded data at the onset of the stimulus. The possible reason is that the electrical activity in each spatial pixel is time-varying for moving stimuli. To quickly elevate neural activity to its peak, the initial values in the GL_DM model were trained to be higher, resulting in peak values that are closer to the actual values later. In contrast, the initial values in the GLM model matched the actual values, but its peak values were lower than the actual values. The goodness of fit for the two sets of data is shown in Figure 4c,d. It is observed that the GL_DM model exhibits a goodness of fit closer to one, indicating a better fit. Additionally, there is a significant difference in the goodness of fit between the two models (*p* < 0.001, paired *t*-test). This result suggests that employing nonlinear functions instead of linear computations to describe peripheral modulation processes leads to more accurate prediction of neuronal responses. To some extent, this validates that the dynamic nonlinear stimulus integration method is more consistent with the information processing of tectal neurons.

**Figure 5:**
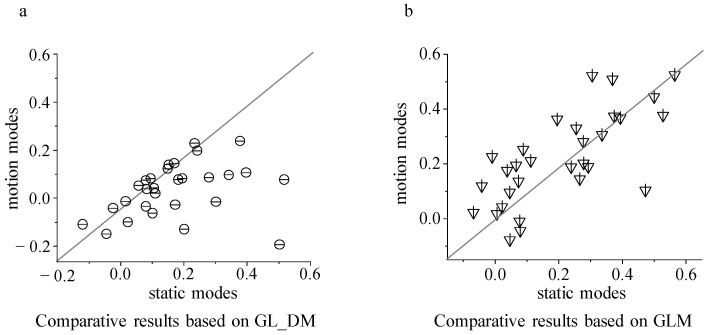
The comparative results of luminance modulation index between static and motion modes. (**a**) Luminance modulation index between static and motion modes calculated from the GL_DM model; (**b**) luminance modulation index between static and motion modes calculated from the GLM model.

Based on the neuronal responses (30 trials) predicted by the two encoding models, the luminance contrast modulation index was calculated for static and motion modes, respectively (Figure 5).

By comparing the modulation index corresponding to the brightness contrast stimulus in static and motion modes, it was found that the GL_DM model can significantly characterize the dynamic modulation rules in static and motion modes (*p* < 0.001, paired *t*-test), while for the static model calculated based on the GLM model there was no significant difference between the brightness contrast modulation index and the motion mode (*p* > 0.5, paired *t*-test). Combining the results shown in Figure 4 and Figure 5, it can be seen that both models can describe the surrounding modulation pattern well in the same stimulation mode (static or moving, Figure 4). However, for the two stimulation modes (Figure 5), the GL_DM model performed well, whereas the GLM model performed poorly. It can be concluded that the dynamic nonlinear stimulus integration has more advantages in characterizing dynamic surrounding modulation and is more consistent with the response characteristics of tectal neurons.

### 3.2. The Role of Inhibitory Synaptic Input in Encoding Contrasting Stimuli

**Figure 6:**
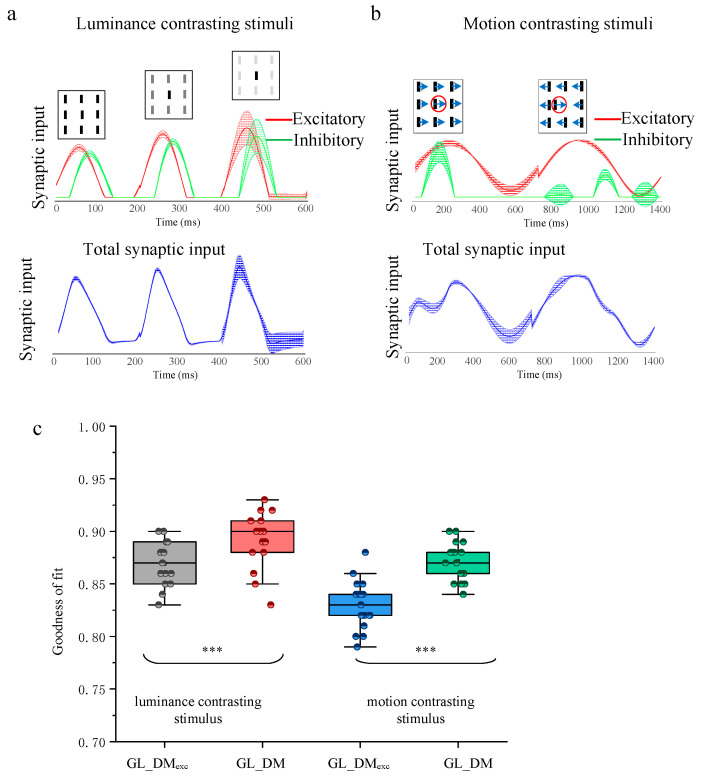
Probing the suppression modulation on spatial luminance and motion direction contrasting stimuli. (**a**) The excitatory synaptic input and inhibitory synaptic input, as well as their corresponding total synaptic input to luminance contrasting stimulus at three contrast levels. (**b**) Similar to (**a**), but to motion contrasting stimulus at two contrast levels. The red circle in each subfigure indicates the receptive field area for the example recording site. The Arrow direction indicates direction of movement. (**c**) The statistical results of GL_DM_exc_ and GL_DM and prediction performance for different levels of contrasting stimuli. The horizontal line indicates the median of each group of data, and the whiskers indicate the lowest and highest points within 1.5× the interquartile ranges of the lower or upper quartile, respectively. The “***” indicates a significant difference between the two groups of data (Wilcoxon signed-rank test, *p* < 0.001).

To gain insight into the prediction performance on the difference between responses to spatial luminance contrasting and those to moving direction contrasting stimuli, we compared the synaptic input of GL_DM (including excitatory synaptic input and inhibitory synaptic input, as well as total synaptic input) to the different contrasting stimuli. The graphs at the top in Figure 6a,b present the excitatory (red) and inhibitory synaptic inputs (green) of GL_DM, and the graphs at the bottom in Figure 6a,b present the total synaptic input (blue) of GL_DM under 20 repeats. Note that the total synaptic input is calculated by passing through the nonlinearities and post-spike filter, so it is not just the direct summation of excitatory and inhibitory synaptic inputs. As the contrast of luminance between the center and the surroundings became larger, all synaptic input increased (Figure 6a). The motion direction contrasting stimuli that the surrounding bars move in the opposite direction induced a stronger excitatory synaptic input and a weaker inhibitory synaptic input than the motion direction uniform stimuli (Figure 6b). So, excitatory synaptic input was positively correlated with the motion direction contrasting index, while inhibitory synaptic input was anti-correlated with the motion direction contrasting index. It is worth noting that the inhibitory synaptic input derived from motion direction contrasting was stronger than that from static luminance contrasting in homogeneous visual contexts, which reflects a stronger modulation induced by motion direction contrasting stimuli compared to spatial luminance contrasting ones.

Finally, we refit the GL_DM with only excitatory synaptic input (GL_DM_exc_) to evaluate the contributions of inhibitory synaptic input to model performance. GL_DM model prediction accuracy was compared to that of GL_DM_exc_ (Figure 6c). Compared to GL_DM_exc_, the GL_DM presented an increased performance (*p* < 0.001, Wilcoxon signed-rank test) compared to the GL_DM_exc_ in predictive response to both spatial luminance (89 ± 2.8% vs. 87 ± 2.1%, *n* = 15) and motion direction contrasting stimuli (87 ± 1.7% vs. 83 ± 2.2%, *n* = 19). This indicated that the full GL_DM achieves superior prediction performance over the GL_DM_exc_ with an inhibitory synaptic input. Furthermore, compared to GL_DM, the GL_DM_exc_ exhibits large reductions (mean reduction: 5%) in predictions of response to direction contrasting stimuli, far greater than in predictions of response to luminance contrasting stimuli (mean reduction: 2%). This further suggests that it is associated with the suppression modulation phenomenon that the response of tectal neurons was modulated more intensely by motion direction than by spatial luminance.

## 4. Discussion

In this study, we derived a contrasting encoding model for tectal neurons using multi-unit recording data. Data were used to fit model parameters and evaluate different strategies of the tectal neuron encoding luminance and motion contrasting stimuli. The results show that the predicted response is more accurate when using GL_DM with a nonlinear function than GLM_D_ with a linear one, verifying the nonlinearity of integration between excitatory and inhibitory inputs to encoding contrasting stimuli. Furthermore, compared to GL_DM, the GL_DM_exc_ exhibited far larger reductions in prediction response to motion direction contrasting stimuli than that to luminance contrasting stimuli. In summary, these results indicated that tectal neurons may adopt different strategies for encoding luminance and motion contrasting stimuli, and the encoding strategies are closely associated with nonlinear integration and inhibitory synaptic input. This provides some explanation as to how and why neuronal responses to motion contrasting and luminance contrasting stimuli differ, providing new insights for understanding the encoding mechanisms of contrasting stimuli based on center–surround suppression in the visual system.

Nonlinear integration mechanisms have been widely found in mammals [27]. Previous studies have demonstrated that the nonlinear relationship between excitatory activation and spike response could be changed by the activation of suppressive subunits [28]. This nonlinear relationship of subunits in the RF center is important for capturing responses to spatially structured stimuli [29]. In addition, theoretical studies have proved the necessity of nonlinearity, such as rectification, followed by a summation of the preprocessed signal [30]. In our study, we found that GL_DM with nonlinear integration maintained more accurate predictive performance than the GLM_D_, which has only one stimulus filter in which synaptic excitation and inhibition are linearly governed by equal filters of opposite signs. However, the GL_DM’s greater accuracy in predicting neuronal responses is attributable to the flexibility conferred by the slight differences in these filters with separate nonlinearities. The results were in agreement with those found in mammals [30,31]. Indeed, the center–surround arrangement of receptive fields is ideal for detecting local contrast changes, and nonlinear spatial integration significantly enhanced surround suppression.

The center–surround suppression may be closely related to information received by neurons, including from feedforward, horizontal, and feedback connections [32,33]. Indeed, the OT is a multi-stratified structure and contains 15 alternating cell and fiber layers [34,35]. It is divided into two functional subdivisions. One is the visual subdivision (OT_v_), which comprises the superficial layers of OT, mainly receiving input from the retina afferents [36], and the other is the multimodal [37] and motor [38] subdivision (OT_id_), which comprises the deeper OT layers [39], dominated by feedback axons from the isthmic system [40]. Our recordings were in the intermediate and deep layers of the pigeon optic tectum (OT_id_) and may be mediated by the network of inhibition from the isthmic system. In birds, the network of inhibitions has been described thoroughly. Inputs from isthmic nucleus interacted divisively with inputs from inside the RF. These interactions enabled powerful surround suppression and could even eliminate neuronal responses to stimuli within the RF, without changing the unit’s tuning for stimuli [41]. It has recently been suggested that cue–invariant motion sensitivity could be mediated by cellular rather than network mechanisms [42]. However, it is of note that this interpretation does not exclude tectal networks with synaptic depression that may mediate tectal responses to more complex dynamic spatiotemporal stimuli such as relative motion. After all, synaptic depression can endow networks with new and unexpected dynamic properties [42]. Our results show that the stronger modulation induced by motion direction than that by spatial luminance may stem from a feedback inhibitory mechanism. We, therefore, guessed that the modulation from luminance contrasting and motion contrasting might be closely related to the feedback of the isthmic system.

Altogether, our results have shown two mechanisms involved in center–surround suppression of contrast for static luminance and motion direction. One is nonlinearly integrated, and the other is suppression modulation. These findings highlight the diversity of center–surround integration as a crucial component for understanding visual encoding in complex dynamic stimuli. The next challenge would be to unravel more mechanisms and properly incorporate them into the model, helping us to comprehensively reveal the encoding strategies of contrasting stimuli. For example, we will incorporate a spatiotemporal information accumulation computation mechanism to improve the GL_DM model’s accuracy at the onset of the stimulus.

## 5. Conclusions

This study investigated the impact of dynamic nonlinear spatial integration on encoding contrasting stimuli in tectal neurons using computational models. We found that dynamic nonlinear spatial integration plays a crucial role in information representation. Additionally, inhibitory synaptic input is implicated in modulating tectal neuron responses, particularly in the suppression modulation phenomenon. These findings shed light on the neural mechanisms involved in target detection during animal behaviors such as foraging and predator avoidance. However, a limitation of our study is the finite number of neurons examined, warranting future research with larger datasets.

## Data Availability

The datasets analyzed during the current study are available from the corresponding author upon reasonable request.

## References

[1] Tadin D. (2022). Strong evidence against a common center-surround mechanism in visual processing. J. Vis..

[2] Turner M.H., Schwartz G.W., Rieke F. (2018). Receptive field center-surround interactions mediate context-dependent spatial contrast encoding in the retina. eLife.

[3] Cui Y., Wang Y.V., Park S.J., Demb J.B., Butts D.A. (2016). Divisive suppression explains high-precision firing and contrast adaptation in retinal ganglion cells. eLife.

[4] Barchini J., Shi X., Chen H., Cang J. (2018). Bidirectional encoding of motion contrast in the mouse superior colliculus. eLife.

[5] Fisher T.G., Alitto H.J., Usrey W.M. (2017). Retinal and Nonretinal Contributions to Extraclassical Surround Suppression in the Lateral Geniculate Nucleus. J. Neurosci. Off. J. Soc. Neurosci..

[6] Henry C.A., Jazayeri M., Shapley R.M., Hawken M.J. (2020). Distinct spatiotemporal mechanisms underlie extra-classical receptive field modulation in macaque V1 microcircuits. eLife.

[7] Wang T., Li Y., Yang G., Dai W., Yang Y., Han C., Wang X., Zhang Y., Xing D. (2020). Laminar Subnetworks of Response Suppression in Macaque Primary Visual Cortex. J. Neurosci..

[8] Gu Y., Wang Y., Wang S. (2000). Regional variation in receptive field properties of tectal neurons in pigeons. Brain Behav. Evol..

[9] Wang Y., Xiao J., Wang S.R. (2000). Excitatory and inhibitory receptive fields of tectal cells are differentially modified by magnocellular and parvocellular divisions of the pigeon nucleus isthmi. J. Comp. Physiol. A.

[10] Niu X., Huang S., Zhu M., Wang Z., Shi L. (2022). Surround Modulation Properties of Tectal Neurons in Pigeons Characterized by Moving and Flashed Stimuli. Animals.

[11] Harmening W.M., Wagner H. (2011). From optics to attention: Visual perception in barn owls. J. Comp. Physiol. A.

[12] Niu X., Huang S., Yang S., Wang Z., Li Z., Shi L. (2020). Comparison of pop-out responses to luminance and motion contrasting stimuli of tectal neurons in pigeons. Brain Res..

[13] Ben-Tov M., Donchin O., Ben-Shahar O., Segev R. (2015). Pop-out in visual search of moving targets in the archer fish. Nat. Commun..

[14] Coen-Cagli R., Kohn A., Schwartz O. (2015). Flexible gating of contextual influences in natural vision. Nat. Neurosci..

[15] Cafaro J., Rieke F. (2013). Regulation of Spatial Selectivity by Crossover Inhibition. J. Neurosci. Off. J. Soc. Neurosci..

[16] Trong P.K., Rieke F. (2008). Origin of correlated activity between parasol retinal ganglion cells. Nat. Neurosci..

[17] Weber A.I., Pillow J.W. (2017). Capturing the Dynamical Repertoire of Single Neurons with Generalized Linear Models. Neural Comput..

[18] Pillow J.W., Paninski L., Uzzell V.J., Simoncelli E.P., Chichilnisky E.J. (2005). Prediction and decoding of retinal ganglion cell responses with a probabilistic spiking model. J. Neurosci. Off. J. Soc. Neurosci..

[19] Rodieck R.W. (1965). Quantitative analysis of cat retinal ganglion cell response to visual stimuli. Vis. Res..

[20] Matzner A., Gorodetski L., Korngreen A., Bar-Gad I. (2020). Dynamic input-dependent encoding of individual basal ganglia neurons. Sci. Rep..

[21] Latimer K.W., Fairhall A.L. (2020). Capturing Multiple Timescales of Adaptation to Second-Order Statistics With Generalized Linear Models: Gain Scaling and Fractional Differentiation. Front. Syst. Neurosci..

[22] Lin Y.S., Chen C.C., Greenlee M.W. (2022). The role of lateral modulation in orientation-specific adaptation effect. J. Vis..

[23] Faunes M., Fernandez S., Gutierrez-Ibanez C., Iwaniuk A.N., Wylie D.R., Mpodozis J., Karten H.J., Marin G. (2013). Laminar segregation of GABAergic neurons in the avian nucleus isthmi pars magnocellularis: A retrograde tracer and comparative study. J. Comp. Neurol..

[24] Latimer K.W., Rieke F., Pillow J.W. (2019). Inferring synaptic inputs from spikes with a conductance-based neural encoding model. eLife.

[25] Luksch H., Karten H.J., Kleinfeld D., Wessel R. (2001). Chattering and differential signal processing in identified motion-sensitive neurons of parallel visual pathways in the chick tectum. J. Neurosci. Off. J. Soc. Neurosci..

[26] Lai D., Brandt S., Luksch H., Wessel R. (2011). Recurrent antitopographic inhibition mediates competitive stimulus selection in an attention network. J. Neurophysiol..

[27] Ito S., Si Y., Litke A.M., Feldheim D.A. (2021). Nonlinear visuoauditory integration in the mouse superior colliculus. PLoS Comput. Biol..

[28] Rust N.C., Schwartz O., Movshon J.A., Simoncelli E.P. (2005). Spatiotemporal Elements of Macaque V1 Receptive Fields. Neuron.

[29] Freeman J., Field G.D., Li P.H., Greschner M., Gunning D.E., Mathieson K., Sher A., Litke A.M., Paninski L., Simoncelli E.P. (2015). Mapping nonlinear receptive field structure in primate retina at single cone resolution. eLife.

[30] Karamanlis D., Gollisch T. (2021). Nonlinear Spatial Integration Underlies the Diversity of Retinal Ganglion Cell Responses to Natural Images. J. Neurosci. Off. J. Soc. Neurosci..

[31] Li Y., Young L.S. (2021). Unraveling the mechanisms of surround suppression in early visual processing. PLoS Comput. Biol..

[32] Pan X., DeForge A., Schwartz O. (2023). Generalizing biological surround suppression based on center surround similarity via deep neural network models. PLoS Comput. Biol..

[33] Li Y., Dai W.F., Wang T., Wu Y.J., Dou F., Xing D.J. (2023). Visual surround suppression at the neural and perceptual levels. Cogn. Neurodynamics.

[34] Luksch H. (2003). Cytoarchitecture of the avian optic tectum: Neuronal substrate for cellular computation. Rev. Neurosci..

[35] Wang Y., Luksch H., Brecha N.C., Karten H.J. (2006). Columnar projections from the cholinergic nucleus isthmi to the optic tectum in chicks (Gallus gallus): A possible substrate for synchronizing tectal channels. J. Comp. Neurol..

[36] Hamdi F.A., Whitteridge D. (1954). The representation of the retina on the optic tectum of the pigeon. Q. J. Exp. Physiol. Cogn. Med. Sci..

[37] Weigel S., Kuenzel T., Lischka K., Huang G., Luksch H. (2022). Morphology and dendrite-specific synaptic properties of midbrain neurons shape multimodal integration. J. Neurosci. Off. J. Soc. Neurosci..

[38] Wang S., Ma Q., Qian L., Zhao M., Wang Z., Shi L. (2022). Encoding Model for Continuous Motion-sensitive Neurons in the Intermediate and Deep Layers of the Pigeon Optic Tectum. Neuroscience.

[39] Gonzalez-Cabrera C., Garrido-Charad F., Mpodozis J., Bolam J.P., Marin G.J. (2016). Axon terminals from the nucleus isthmi pars parvocellularis control the ascending retinotectofugal output through direct synaptic contact with tectal ganglion cell dendrites. J. Comp. Neurol..

[40] Marin G., Mpodozis J., Sentis E., Ossandon T., Letelier J.C. (2005). Oscillatory bursts in the optic tectum of birds represent re-entrant signals from the nucleus isthmi pars parvocellularis. J. Neurosci. Off. J. Soc. Neurosci..

[41] Mysore S., Asadollahi A., Knudsen E. (2010). Global Inhibition and Stimulus Competition in the Owl Optic Tectum. J. Neurosci. Off. J. Soc. Neurosci..

[42] Luksch H., Khanbabaie R., Wessel R. (2004). Synaptic dynamics mediate sensitivity to motion independent of stimulus details. Nat. Neurosci..

